# Transient p53/p21 activation selectively protects healthy human hair follicles and their stem cells from chemotherapy

**DOI:** 10.1172/JCI174447

**Published:** 2026-05-01

**Authors:** Jennifer Gherardini, Tara Samra, Tatiana Gomez-Gomez, Aysun Akhundlu, Samantha D. Verling, Kinga Linowiecka, Tongyu C. Wikramanayake, Ulrich Knie, Jose Rodríguez-Feliz, Ramtin Kassir, Wolfgang Funk, Reza P. Azar, D. Allen Annis, Manuel Aivado, Jérémy Chéret, Ralf Paus

**Affiliations:** 1 CUTANEON – Skin & Hair Innovations, Hamburg, Germany.; 2 Dr. Phillip Frost Department of Dermatology & Cutaneous Surgery, University of Miami Miller School of Medicine, Miami, Florida, USA.; 3 Department of Human Biology, Nicolaus Copernicus University, Toruń, Poland.; 4 Jose Rodríguez-Feliz, MD, Soléstica Plastic Surgery & MedSpa, Coral Gables, Florida, USA.; 5 Kassir Plastic Surgery, New York, New York, USA.; 6 Schönheitsklinik Dr Funk München, Munich, Germany.; 7 Zentrum für Moderne Haartransplantation Berlin, Berlin, Germany.; 8 Aileron Therapeutics, Inc., Boston, Massachusetts, USA.

**Keywords:** Dermatology, Stem cells, Apoptosis, Cell cycle, Microcirculation

## Abstract

Chemotherapy-induced alopecia (CIA) remains one of the most distressing adverse effects of cancer therapy. Yet, no therapy is available to selectively protect healthy hair follicles (HFs) and their epithelial stem cells (eHFSCs) from chemotherapy-induced damage without awarding potential survival benefits to cancer cells. Here, we report how human HFs can be protected against 2 lead CIA-inducing chemotherapeutics by inducing selective transient cell cycle arrest. Pretreating scalp HFs before chemotherapy exposure ex vivo with ALRN-6924, a clinical-stage “stapled peptide” drug that binds with high affinity to key endogenous inhibitors of p53, selectively activated p53 signaling only in cells with wild-type TP53 genotype and upregulated p21. This led to temporary cell cycle arrest in healthy tissues without protecting TP53-mutant cancer cells and mitigated chemotherapy-induced HF damage on multiple levels, including excessive hair matrix apoptosis, premature catagen, pigmentary abnormalities, “mitotic catastrophe,” and micronucleation. It also protected eHFSCs against DNA damage, apoptosis, and pathological epithelial-mesenchymal transition. Notably, even topically applied ALRN-6924 afforded relative chemotherapy protection ex vivo. These results provide proof of principle for a strategy to selectively protect rapidly proliferating healthy epithelial tissues and their stem cells in patients with TP53-mutant cancers, which promises to protect against acute and permanent CIA.

## Abstract 2

A novel approach for protecting healthy hair follicles from chemotherapy-induced hair loss, via activating p53/p21 signal in p53 wildtype cells, without protecting p53 mutant cancer cells.

## Introduction

Chemotherapy-induced alopecia (CIA) is one of the most distressing adverse effects patients with cancer experience (1–3). Typically, CIA is reversible after discontinuation of cancer therapy, since chemotherapy-treated hair follicles (HFs) undergo distinctive damage responses that are either reparative (“dystrophic anagen” pathway) or regenerative (“dystrophic catagen” pathway) (4, 5). These responses can be pharmacologically manipulated in mice in vivo or in human HFs ex vivo by administering, for example, steroid hormones, immunophilin ligands, alpha-melanocyte-stimulating hormone, erythropoietin, melatonin, or low-intensity ultrasound (6–12).

Chemotherapy damages rapidly proliferating cells, both cancer cells and normal tissues, such as bone marrow, gut, and — particularly prominently — massively proliferating hair matrix keratinocytes (4, 13, 14). The latter are the key target cell population for acute CIA (4, 11, 15) and undergo p53-dependent apoptosis under chemotherapy (11), which is effected by the downregulation of Shh activity in hair matrix keratinocytes (16). Interestingly, temporary cell cycle arrest by palbociclib, a CDK4/6 inhibitor, can protect hair matrix keratinocytes from chemotherapy-induced damage ex vivo (15). However, there are still no pharmacological interventions available that reliably and selectively protect healthy human HFs cells from chemotherapy-induced damage without simultaneously protecting tumor cells (9, 10, 17).

While the application of scalp cooling systems during chemotherapy infusion can reduce CIA in many patients (18–21), concerns remain that reducing scalp perfusion and tissue sensitivity to chemotherapy by lowering skin temperature might also favor the escape of isolated scalp micrometastases from chemotherapy (4, 22, 23). The same concern applies to temporary cell cycle arrest by CDK4/6 inhibitors (15) and other candidate chemoprotectants that do not differentiate between healthy HF and cancer cells.

There has also been an alarming increase in the incidence of long-term, irreversible hair loss (permanent CIA), defined as incomplete hair regrowth 6 months after cessation of chemotherapy (24), namely after the administration of chemotherapeutic regimens that include the taxanes, paclitaxel (PTX) or docetaxel (24–29), notably in combination with cyclophosphamide, one of the most frequent and best-studied CIA-inducing chemotherapeutics (5, 11, 30). For example, more than 10% of PTX-treated and 23% of docetaxel-treated patients undergoing chemotherapy for gynecological tumors reportedly suffer permanent CIA (28, 31, 32).

Complete hair growth restoration after chemotherapy is only possible if epithelial stem cells in the HF’s bulge region (eHFSCs) survive and remain functional (4, 24, 33). These eHFSCs suffer much more damage than previously suspected from exposure to 2 lead CIA-inducing chemotherapeutics, PTX (15) and the toxic cyclophosphamide metabolite, 4-hydroxycyclophosphamide (4-HC) (30). Ex vivo, this damage ranges from the induction of excessive apoptosis and “mitotic catastrophe” to substantial DNA damage, micronucleation, and even pathological epithelial-mesenchymal transition (EMT) in Keratin 15^+^ eHFSCs (15, 30). Since the human HF’s capacity to renew and replenish its core stem cell populations appears to be quite limited (34, 35), to reduce the risk of permanent CIA, irreversible eHFSC damage must be prevented (4, 33). While CDK4/6 inhibitors, agonistic PPARγ modulators, and melatonin can greatly limit chemotherapy-induced eHFSC damage ex vivo (12, 15, 30), cancer cells might also profit from these candidate eHFSC chemoprotectants.

Therefore, it remains a major and pressing unmet medical need to develop effective pharmacological strategies that selectively protect healthy human HFs and their eHFSCs from acute and long-term chemotherapy-induced damage leading to acute or permanent CIA. Ideally, such drugs should be applied topically to further reduce the risk that extracutaneous cancer cells profit from chemoprotection. Clinical availability of such chemoprotectants would drastically improve the quality of life of cancer patients with CIA (36–38) and may also reduce the number of patients refusing lifesaving chemotherapy for fear of suffering CIA (29, 39).

To address this unmet medical need, we turned to ALRN-6924, a clinical-stage “stapled peptide” drug that mimics the N-terminal domain of the p53 tumor suppressor protein (40–42). ALRN-6924 is negatively charged, which facilitates cell uptake via macropinocytosis, a regulated form of endocytosis that drives the nonselective uptake of solute molecules (40, 43). Subsequent intracellular proteolysis of ALRN-6924 forms a long-acting active metabolite (40) with potent binding affinity to MDM2, and MDMX, 2 key endogenous inhibitors of p53 (44). This activates p53 signaling selectively in cells having a wild-type TP53 genotype (TP53-WT) (41, 45, 46).

At high doses, ALRN-6924 exhibits anticancer activity, while at lower doses, ALRN-6924 transiently arrests the cell cycle in healthy cells with TP53-WT via p21 upregulation, thus selectively protecting them from chemotherapy without protecting TP53-mutant cancer cells (40, 47–49), as documented, e.g., in healthy, normal TP53-WT bone marrow and gastrointestinal cells (40, 42). In clinical studies, ALRN-6924 protects patients with small cell lung cancer from topotecan-induced myelosuppression without negatively affecting the tumor response to topotecan in patients with TP53-mutant tumors (50, 51). Since roughly half of all cancers carry p53 mutations (52–54), including gynecological and breast cancers treated most frequently with taxanes or cyclophosphamide (55, 56), a vast population of chemotherapy-treated patients with TP53-mutant tumors might, in principle, benefit from this selective chemoprotection strategy and thus avoid or at least reduce CIA.

Therefore, the current study aimed to determine whether selective, p53/p21-dependent temporary cell cycle arrest by ALRN-6924 only in normal, TP53-WT cells is indeed chemoprotective in highly chemotherapy-vulnerable human scalp HFs (2, 4, 32). This was explored after “systemic” ALRN-6924 addition to the medium of microdissected, full-length human scalp HFs or full-thickness human scalp skin, and after topical ALRN-6924 application to full-thickness human scalp skin, using our well-established ex vivo assays (15, 30, 57).

## Results

### p53 activation induces intrafollicular cell cycle arrest without exerting HF toxicity or promoting catagen.

**Figure 1:**
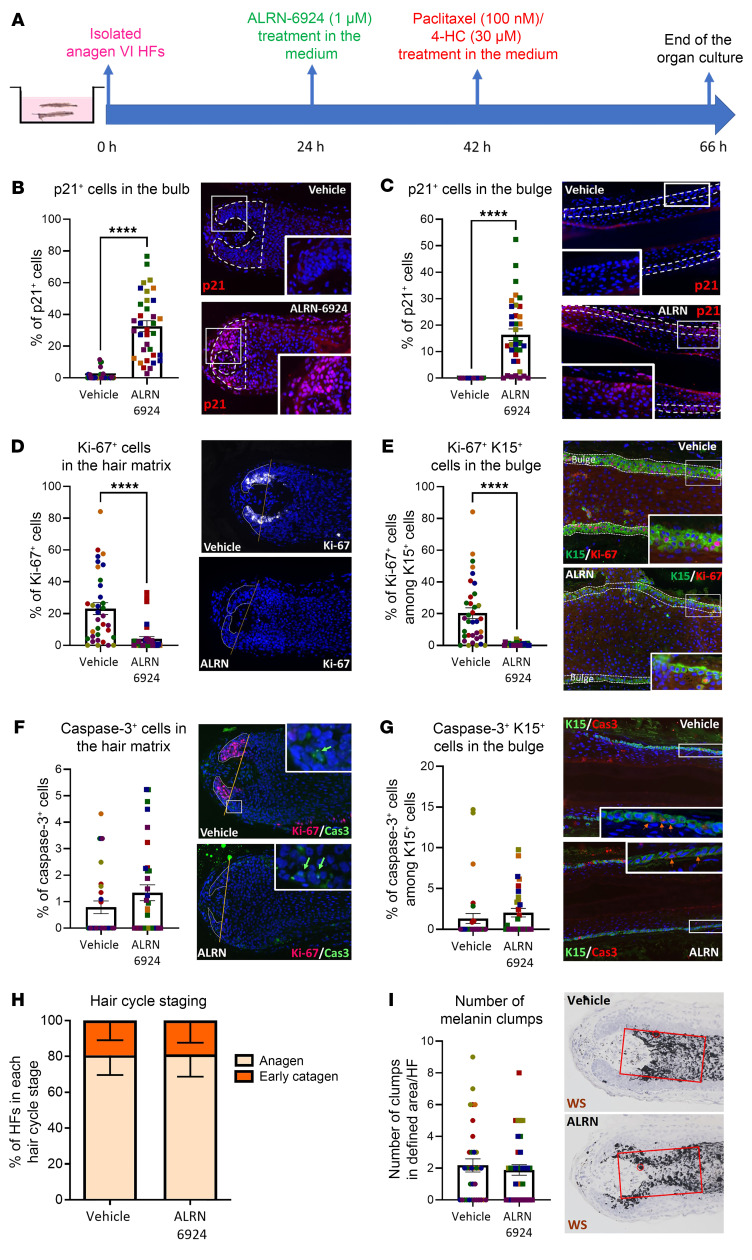
Effect of ALRN-6924 on organ-cultured, microdissected human HFs. (**A**) Experimental design scheme. (**B**–**I**) Quantitative (immuno-)histomorphometry and representative images showing cell cycle arrest mediated by ALRN-6924. (**B** and **C**) Percentage of p21^+^ cells in the hair matrix and (**B**) in the bulge (**C**). (**D** and **E**) Percentage of Ki-67^+^ cells in the hair matrix tips (**D**) and percentage of Ki-67^+^ cells among the total number of K15^+^ in the bulge (**E**). (**F** and **G**) Percentage of caspase-3^+^ cells in the hair matrix (**F**) and caspase-3^+^ cells among the total number of K15^+^ in the bulge (**G**). (**H**) Hair cycle staging showing the percentage of HFs in each stage. (**I**) Number of melanin clumps in the defined reference area. Mean ± SEM; *N* = 20–29 HF/group from 6 donors. Student’s *t* test, ****P < 0.0001. Green and orange arrows: caspase-3^+^ cells. WS, Warthin-Starry.

First, we asked whether ALRN-6924 alone induces the expected upregulation of p21 as an indicator of p53-induced cell cycle arrest (45, 58) in the hair matrix and bulge epithelium of organ-cultured full-length human scalp HF (15, 30), using the experimental protocol summarized in Figure 1A and Supplemental Figure 1A; supplemental material available online with this article; https://doi.org/10.1172/JCI174447DS1 Briefly, HFs were pretreated with either vehicle (medium alone) or 1 μM ALRN-6924, known to induce p21 expression and cell cycle arrest with minimal apoptosis (40, 42). After 18 hours, the medium was replaced with either vehicle or 1 μM ALRN-6924 (on systemic administration in vivo, the ALRN-6924 effect is temporary, reaching its maximum after 18 hours, after which cells restart their progression into the cell cycle as ALRN-6924 is cleared from circulation) (50). Then, 24 hours later, HFs were snap-frozen and processed. To mimic the clinical situation as closely as possible ex vivo, we did not synchronize the cell cycle in these studies and used only a relatively short exposure time to ALRN-6924.

Quantitative (immuno-)histomorphometry (qIHM) of p21^+^ cells showed that ALRN-6924 significantly enhanced p21 protein expression in both the anagen hair matrix (Figure 1B) and the bulge epithelium (Figure 1C) of healthy human HFs, as expected. That not all cells were positive for p21 suggests that not all cells were in the same cell cycle stage during treatment, as supported by the fact that less than 100% of hair bulb cells were Ki-67^+^. The ALRN-6924–induced enhanced p21 expression corresponded to a significant reduction in the fraction of proliferating (Ki-67^+^) cells in the hair matrix (Figure 1D) and of Keratin 15^+^ (K15^+^) eHFSCs in the bulge (59, 60) (Figure 1E) compared with vehicle-treated control HFs. As expected of low-dose ALRN-6924 (42), the percentage of apoptotic (caspase-3^+^) cells in the hair matrix and bulge did not significantly increase (Figure 1, F and G). ALRN-6924 neither significantly altered the number of K15^+^ eHFSC (Supplemental Figure 1B) nor the overall expression of K15 protein in the HF epithelium (Supplemental Figure 1, C and D) after 3 days of organ culture.

ALRN-6924 alone promoted neither apoptosis-driven HF regression (catagen development) (61, 62) nor HF toxicity. Standardized hair cycle histomorphometry (63) demonstrated that the vast majority (ca. 80%) of both vehicle- or ALRN-6924–treated HFs remained in anagen after 3 days ex vivo (Figure 1H). No significant pigmentary abnormalities (i.e., melanin clumping and ectopic location of melanin granules, a very sensitive HF toxicity indicator) (8, 11, 12) were seen in the hair matrix in ALRN-6924–treated HFs (Figure 1I). This is clinically important since candidate chemoprotectants that are HF toxic and promote catagen development would aggravate acute CIA by promoting the “dystrophic catagen” response pathway of HFs to chemotherapy-induced damage (4, 8, 33). Thus, this MDM2/MDMX-binding peptide robustly induces p53/p21-mediated cell cycle arrest in a healthy human (mini-) organ ex vivo (Figure 1, D and E) without promoting apoptosis, catagen, and HF toxicity or negatively impacting eHFSCs under assay conditions.

### ALRN-6924 reduces chemotherapy-induced damage of the hair matrix and promotes the reparative “dystrophic anagen” pathway.

**Figure 2:**
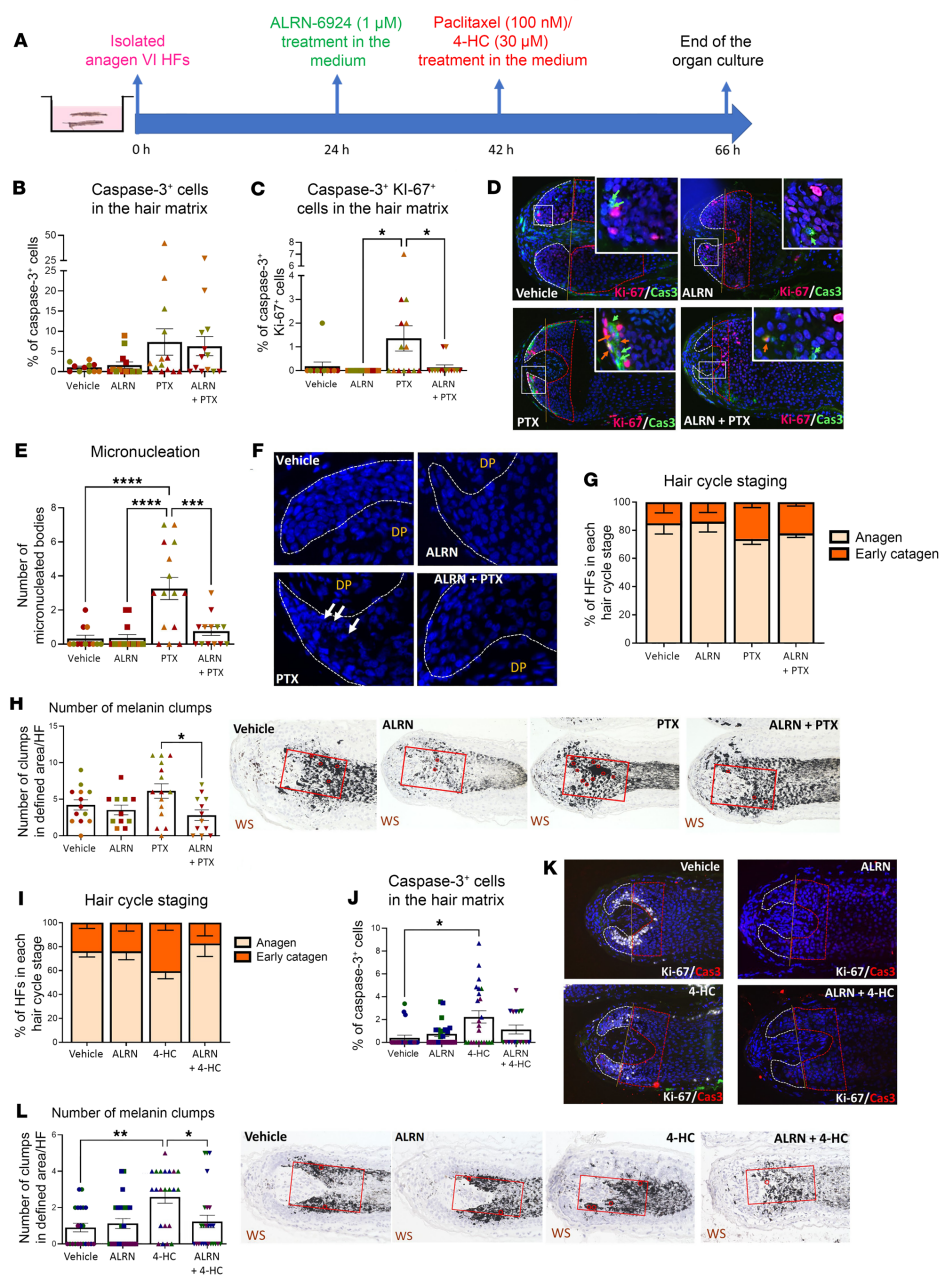
ALRN-6924 reduces PTX- or 4-HC–induced damage of the anagen hair matrix and promotes “dystrophic anagen” in the microdissected HF. (**A**) Experimental design scheme. (**B**–**L**) Quantitative (immuno-)histomorphometry and representative images showing the hair follicle damage induced by PTX and 4-HC and the protective role of ALRN-6924. (**B** and **J**) Percentage of caspase-3^+^ cells in the hair matrix. (**C**) Number of cells in mitotic catastrophe, caspase-3^+^Ki-67^+^ cells in the hair matrix. (**D** and **K**) Representative images of Ki-67/caspase-3 double staining. (**E** and **F**) Number of micronucleated bodies and representative images of DAPI^+^ nuclei. (**G** and **I**) Hair cycle staging showing the percentage of HFs in each stage. (**H** and **L**) Number and representative images of melanin clumps in the defined reference area. Mean ± SEM; *N* = 22–24 HF/group from 3 donors treated with PTX or 4-HC. Student’s *t* test, *P < 0.05, **P < 0.01, ***P < 0.001, ****P < 0.0001. Green arrows: caspase-3^+^ cells; orange arrows: mitotic catastrophe; caspase-3^+^Ki-67^+^ cells; white arrows: micronucleated bodies. Note that the vehicle and ALRN-6924 data in graphs **B**, **G**–**J**, and **L** were used to generate the graphs presented in Figure 1, F, H, and I.

Next, we investigated whether pretreating microdissected, organ-cultured anagen HFs with ALRN-6924 for 18 hours before chemotherapy exposure protected the hair matrix from chemotherapy damage (Figure 2A and Supplemental Figure 2A).

As expected (15), PTX alone induced massive apoptosis (Figure 2B), “mitotic catastrophe” (i.e., simultaneous entry into the cell cycle and activation of the apoptotic machinery) (9, 68–70) (Figure 2, C and D), and micronucleation (Figure 2, E and F) in hair matrix keratinocytes compared with vehicle-treated HFs. Surprisingly, under our assay conditions, PTX did not promote catagen ex vivo (Figure 2G). When microdissected anagen scalp HFs were pretreated with ALRN-6924 to induce cell cycle arrest via p21 (see Figure 1, B and C), PTX-induced hair matrix damage/cytotoxicity was minimized as shown by the significant reduction of HF pigmentary abnormalities (melanin clumping) (Figure 2H), “mitotic catastrophe” (Figure 2, C and D), and micronucleation (Figure 2, E and F) compared with HFs treated only with PTX.

HF treatment with 4-HC (30 μM) induced the expected premature development of catagen (7, 11) (Figure 2I) and significantly increased hair matrix apoptosis (Figure 2, J and K) but did not induce “mitotic catastrophe” (data not shown). Instead, when ALRN-6924 was added to the HF organ culture medium, this “systemic” exposure prevented 4-HC–induced premature catagen development (Figure 2I) as well as enhanced hair matrix apoptosis (Figure 2, J and K) and HF damage/cytotoxicity (Figure 2L).

Furthermore, periodic acid–Schiff (PAS) histochemistry, which visualizes intrafollicular glycogen production and the integrity of the HF’s basement membrane (67–69), showed that PTX and 4-HC both thinned and disrupted the basement membrane around the hair bulb. Unexpectedly, ALRN-6924 prevented this chemotherapy-induced basement membrane damage (see Supplemental Figure 2, B and C), suggesting that this peptide has protective effects beyond cell cycle arrest. Histochemically, the intrafollicular glycogen production (67) remained unaltered (Supplemental Figure 2, B and C). Although both PTX and 4-HC promoted HF toxicity, hair shaft production (Supplemental Figure 2, D and E) and expression of the hair shaft keratin, K85, a sensitive marker for the terminal differentiation of human hair matrix keratinocytes (14, 70), were unaltered (Supplemental Figure 2, F and G). ALRN-6924 showed no impact on hair shaft production or K85 expression in the hair matrix (Supplemental Figure 2, D–G).

**Figure 3:**
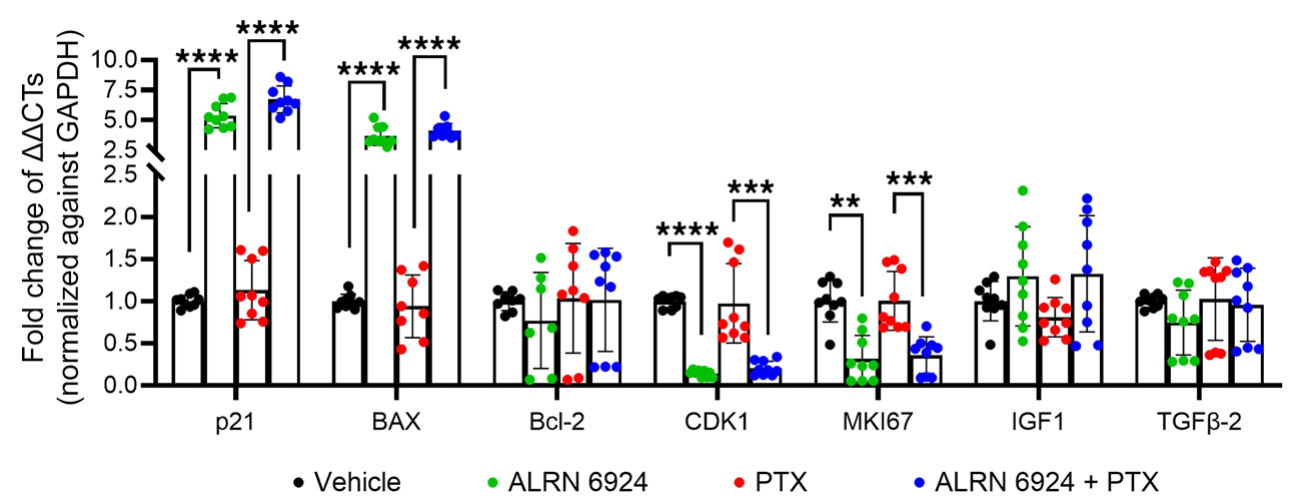
qPCR of HFs treated with ALRN-6924 and/or PTX. qPCR shows that ALRN-6924 stimulates the p53 signaling pathway by promoting p21 and BAX transcription. This led to cell cycle arrest, as indicated by increased transcription for CDK1 and MKI67. The increased BAX levels, along with essentially unaltered Bcl-2 transcript levels, raise the possibility that ALRN-6924 might favor the induction of BAX-dependent apoptosis. Of note, PTX increases TGF-β2, a potent hair growth inhibition factor, indicating that PTX promotes catagen. However, ALRN-6924, when coadministered with PTX, at the timeline tested here, failed to significantly reduce TGF-β2. Finally, ALRN-6924 does not alter the transcription of hair growth factor, IGF-1, indicating that it does not affect hair growth. Mean ± SEM; *N* = 14 HF/group from 3 donors treated with ALRN-6924 and/or PTX. Student’s *t* test, **P < 0.01, ***P < 0.001, ****P < 0.0001.

Finally, we assessed transcription by qRT-PCR of the key anagen-maintenance growth factor, IGF-1 (71), and the main physiological catagen-promoting growth factor, TGF-β2 (72), as well as key downstream targets of p53 signaling, namely Cdkn1a, Bcl-2-associated X protein (BAX), and Nox2 (73, 74). This confirmed cell cycle arrest, as evidenced by the significant decrease in CDK1 and MKI67 steady-state transcript levels, and revealed that the downstream p53 target genes, p21 and BAX, were significantly upregulated in HFs treated with ALRN-6924 (Figure 3). The increased BAX levels, along with essentially unaltered Bcl-2 transcript levels, raise the possibility that ALRN-6924 favors BAX-dependent apoptosis. This is in line with the observation reported below that prolonged ALRN-6924 treatment promotes HF apoptosis. TGF-β2 and IGF-1 gene levels remained unaffected by ALRN-6924 or PTX (Figure 3).

Taken together, these data support that the hair growth–protective effects of ALRN-6924 are primarily mediated by cell cycle arrest, rather than by altering the intrafollicular expression of key hair growth–modulatory growth factors, by suppressing PTX-induced apoptosis, or by altering hair-specific keratin production.

### ALRN-6924 reduces chemotherapy-induced damage when “systemically” applied to intact human scalp skin ex vivo.

**Figure 4:**
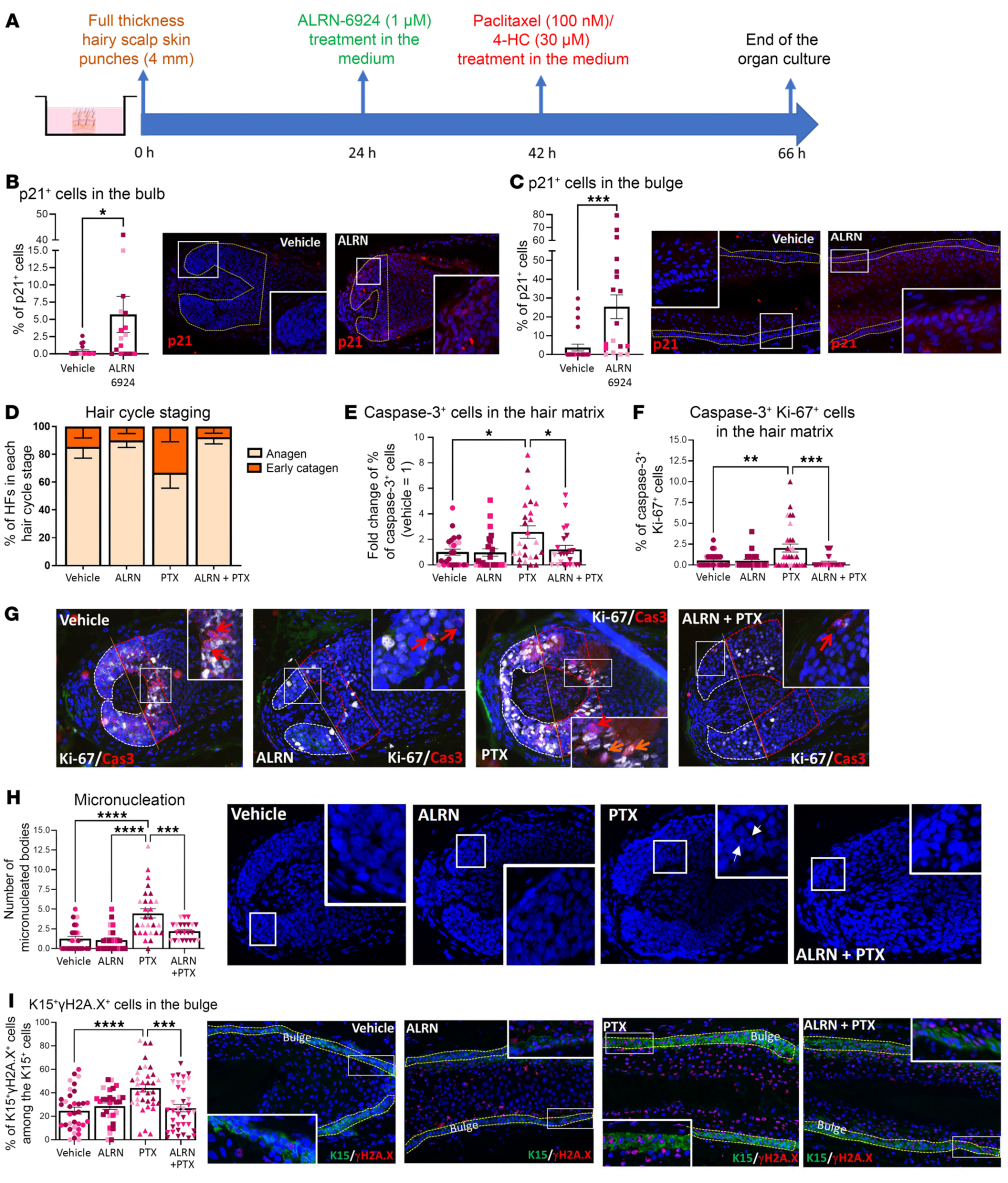
ALRN-6924 promotes dystrophic anagen and protects human HF from PTX-induced hair matrix apoptosis and eHFSC DNA damage in the bulge in full-thickness skin organ culture. (**A**) Experimental design scheme. (**B** and **C**) Percentage of p21^+^ cells in the hair bulb and (**B**) in the bulge (**C**). (**D**) Hair cycle staging showing the percentage of HFs in each stage. (**E**) Percentage of apoptotic (caspase-3^+^) cells in the hair matrix. (**F**) Number of caspase-3^+^Ki-67^+^ cells showing the “mitotic catastrophe” in the hair matrix. (**G**) Representative image of Ki-67/caspase-3 double staining. (**H**) Number of micronucleated bodies and representative images of DAPI^+^ micronucleated bodies. (**I**) Percentage and representative images of γH2A.x-positive cells among the total number of K15^+^ cells in the bulge. Mean ± SEM; *N* = 31–40 HF/group from 3 donors treated with PTX. Student’s *t* test, *P < 0.05, **P < 0.01, ***P < 0.001, ****P < 0.0001. Red arrows: caspase-3^+^ cells; orange arrows: Ki-67^+^caspase-3^+^ cells; white arrows: micronucleated bodies.

Next, we asked whether “systemic” ALRN-6924 treatment also protected HFs in organ-cultured full-thickness human scalp skin from chemotherapy damage (Figure 4A and Supplemental Figure 3A). Again, ALRN-6924 increased the percentage of p21^+^ cells (Figure 4, B and C) and decreased that of Ki-67^+^ cells (Supplemental Figure 3, B and C) in the bulb and bulge and prevented or significantly reduced PTX-induced catagen induction (Figure 4D), apoptosis (Figure 4E), “mitotic catastrophe” (Figure 4, F and G), and micronucleation (Figure 4H) of hair matrix keratinocytes. Thus, ALRN-6924 is chemoprotective also when human HFs remain embedded in their physiological scalp skin habitat. Interestingly, the PTX dose tested did not induce the typical pigmentary signs of hair bulb damage (Supplemental Figure 3D), possibly due to rescue effects exerted, e.g., by HGF-secreting perifollicular dermal white adipose tissue on the HF pigmentary unit (75), which operate in intact scalp skin.

In human scalp HF xenotransplants in vivo, no major cyclophosphamide-induced damage of perifollicular (mouse) blood vessels was reported (76). However, encouraged by work in mice and rats (77, 78), we also investigated the impact of PTX on perifollicular CD31^+^ endothelial cells. qIHM showed a low number of apoptotic endothelial cells (CD31^+^caspase-3^+^) in all experimental groups, revealing no significant differences between vehicle-, chemotherapy-, or PTX + ALRN-6924–treated skin (Supplemental Figure 3E). In line with the literature (8, 11, 15, 16, 76), this suggests that perifollicular blood vessels are not a key target of PTX-induced human HF damage (Supplemental Figure 3, D and E). However, when ALRN-6924 was administered systemically alone ex vivo, the number of CD31^+^caspase-3^+^ cells was significantly increased (Supplemental Figure 3E). Since blood vessels are no longer functional in human skin organ culture (79), the potential contribution of vascular damage induced by ALRN-6924 or PTX to human CIA pathobiology in vivo remains to be definitively clarified.

### ALRN-6924 protects the HF’s epithelial stem cell niche from chemotherapy-induced apoptosis, DNA damage, and pathological EMT.

**Figure 5:**
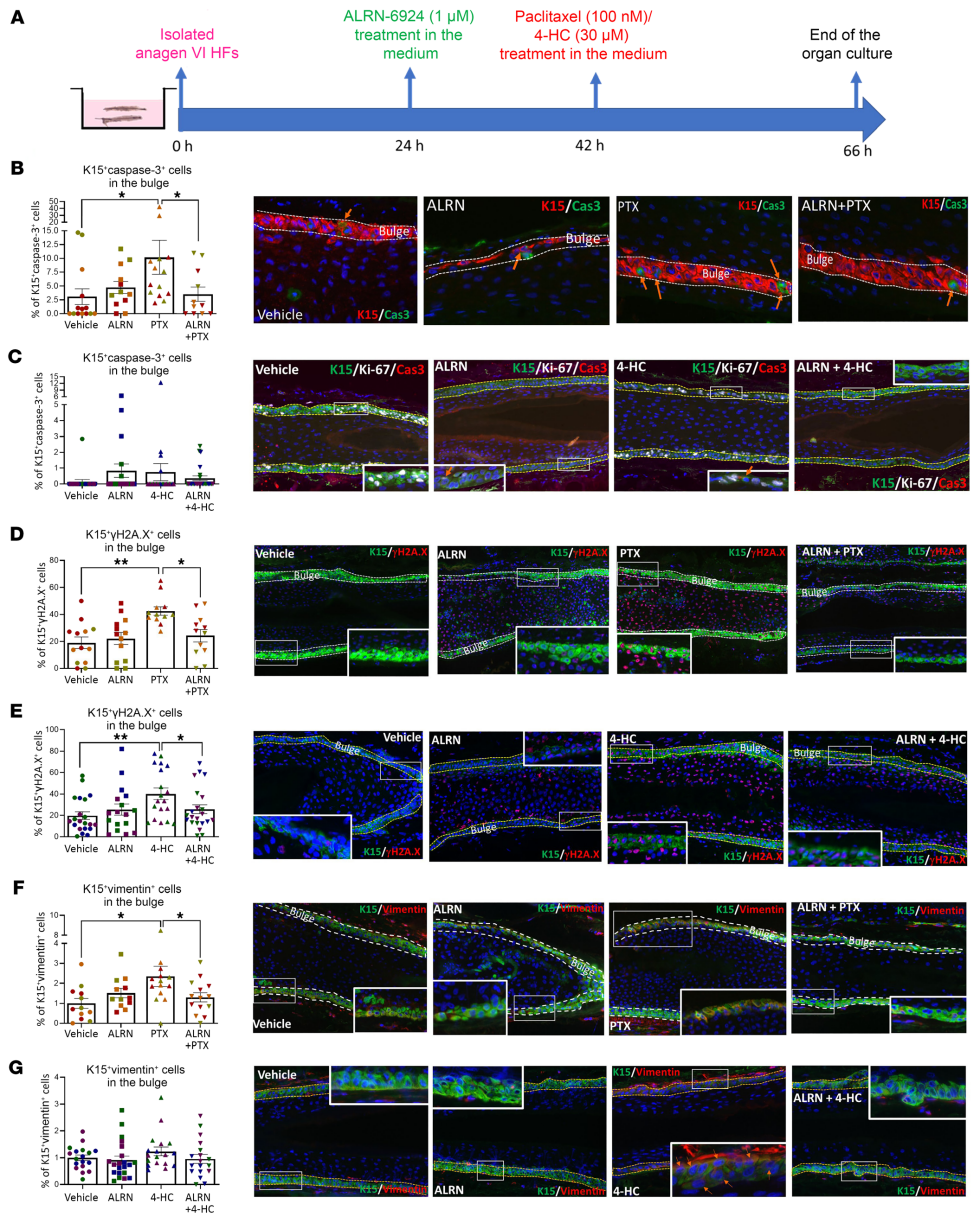
ALRN-6924 protects human eHFSCs from chemotherapy-induced DNA damage and pathological EMT in microdissected HFs. (**A**) Experimental design scheme. (**B**–**G**) Percentage and representative image of apoptotic (caspase-3^+^) (**B** and **C**), γH2A.x-positive (DNA damage marker) (**D** and **E**), and vimentin-positive (marker of pathological EMT) (**F** and **G**) cells among the total number of K15^+^ cells in the bulge. Mean ± SEM; *N* = 18–21 HF/group from 3 donors treated with PTX or 4-HC. Student’s *t* test, *P < 0.05, **P < 0.01. Orange arrows: K15^+^caspase-3^+^ cells. Note that the vehicle and ALRN-6924 data in graphs **D** and **C** were used to generate the graphs presented in Figure 1G.

Since the development of permanent CIA is expected to result from irreversible eHFSC damage (4, 15, 30), we subsequently interrogated chemotherapy-induced bulge damage in organ-cultured, full-length anagen scalp HFs (15, 30) and the effect of “systemic” pretreatment with ALRN-6924 (Figure 5A). qIHM analyses revealed that ALRN-6924 significantly suppressed PTX- and 4-HC–induced apoptosis in K15^+^ eHFSCs compared with HFs pretreated with vehicle, PTX, or 4-HC alone (Figure 5, B and C).

Chemotherapy also induces substantial DNA damage, possibly premature senescence, and even pathological EMT in K15^+^ human eHFSCs ex vivo, thereby compromising the HF’s stem cell–dependent reparative/regenerative capacity in the long term during subsequent hair cycles (4, 15, 30, 33). Indeed, treatment with either PTX (15) or 4-HC alone (30) led to the expected significant increase in the percentage of double-positive γH2A.x^+^K15^+^ (Figure 5, D and E) and vimentin^+^K15^+^ eHFSCs (Figure 5, F and G). This confirms that these chemotherapeutics induce not only apoptosis but also major DNA damage and pathological EMT within the bulge stem cell niche (30, 80) — a persuasive explanation for the frequency of permanent CIA seen in patients treated with taxanes, even long after the termination of chemotherapy (26, 81, 82). Incidentally, this constitutes the first demonstration to our knowledge that PTX can induce pathological EMT in the bulge of human HFs ex vivo.

Importantly, qIHM documented that HF pretreatment with ALRN-6924 significantly reduced DNA damage in K15^+^ eHFSCs (Figure 5D) and pathological EMT induction by PTX (Figure 5F). Very similar bulge DNA damage– and EMT-protective effects were also observed in ALRN-6924–pretreated HFs when 4-HC was subsequently administered (Figure 5, E and G). K15^+^ eHFSCs were protected by ALRN-6924 from PTX-induced DNA damage also in intact, organ-cultured full-thickness scalp skin (Figure 4I). However, the PTX dose investigated failed to induce substantial apoptosis and pathological EMT in eHFSCs in scalp skin organ culture, so apoptosis- and EMT-protective effects of ALRN-6924 were not measurable under these skin organ culture conditions (Supplemental Figure 3, F and G).

Thus, p53/p21 activation by “systemic” ALRN-6924 protects the eHFSC niche of human scalp HFs from chemotherapy-induced damage ex vivo.

### Prolonged exposure to ALRN-6924 is not HF toxic.

**Figure 6:**
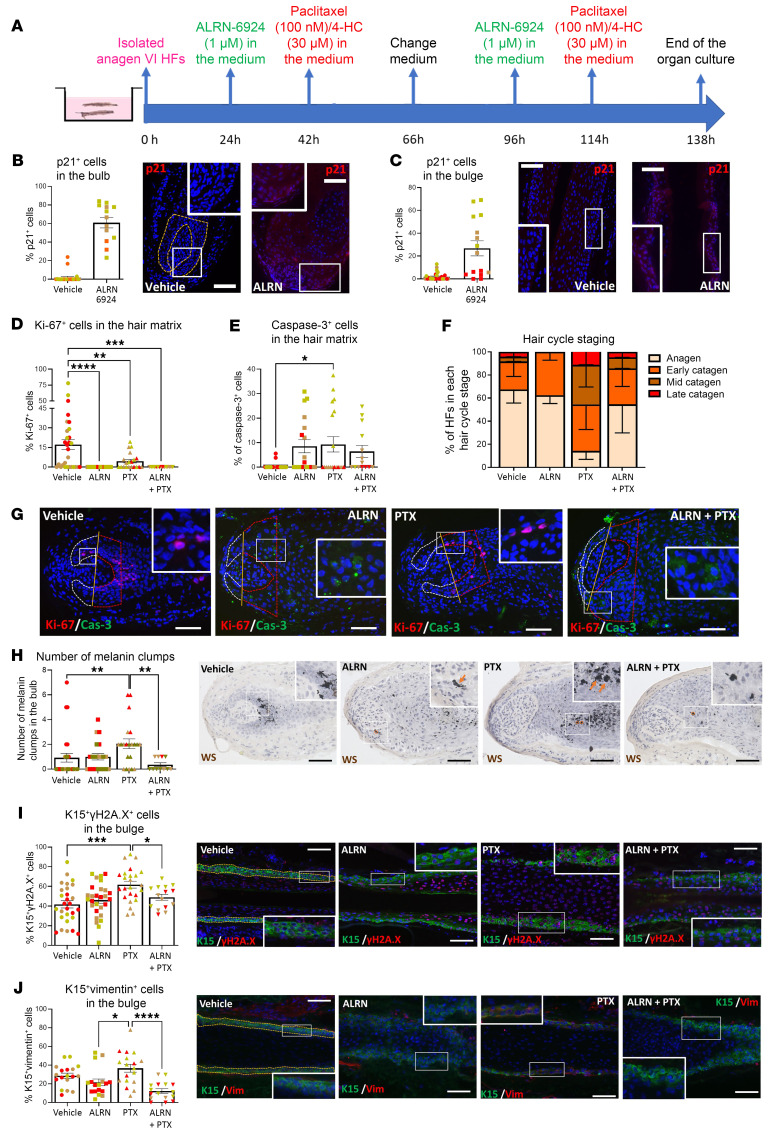
Extended exposure to ALRN-6924 does not promote cytotoxicity and still prevents PTX-induced HF damage. (**A**) Experimental design scheme. (**B**–**J**) Quantitative (immuno-)histomorphometry and representative images showing the hair follicle damage induced by PTX and the protective role of ALRN-6924 after 6 days of organ culture. (**B** and **C**) Percentage of p21^+^ cells in the HF’s bulb (**B**) and bulge (**C**). (**D**) Percentage of caspase-3^+^ cells in the hair matrix. (**E**) Percentage of Ki-67^+^ cells in the hair matrix. (**F**) Hair cycle staging showing the percentage of HFs in each stage. (**G**) Representative images of Ki-67/caspase-3 double staining. (**H**) Number and representative images of melanin clumps in the defined reference area. (**I** and **J**) Percentage and representative image of γH2A.x-positive (DNA damage marker) (**I**) and vimentin-positive (a marker of pathological EMT) (**J**) cells among the total number of K15^+^ cells in the bulge. Mean ± SEM; *N* = 15–26 HF/group from 3 donors treated with PTX or 4-HC. Student’s *t* test, *P < 0.05, **P < 0.01, ***P < 0.001, ****P < 0.0001. Orange arrows: melanin clumps. Scale bar 50 μm.

In order to probe if prolonged cell cycle arrest by ALRN-6924 exerts HF toxicity or pushes anagen HFs prematurely into catagen (15, 63), these were treated twice with ALRN-6924 and/or chemotherapy over 6 days (Figure 6A). Again, long-term administration of ALRN-6924 alone significantly upregulated p21 protein expression in the bulb and bulge (Figure 6, B and C), reduced hair matrix keratinocyte proliferation (Figure 6D), and slightly, but nonsignificantly, promoted apoptosis of hair matrix cells (Figure 6E), as expected from prolonged exposure and intrafollicular accumulation of a cell cycle–arresting peptide. Yet, ALRN-6924 alone promoted neither premature catagen ex vivo (Figure 6, F and G) nor HF cytotoxicity (i.e., no increase in melanin clumping) (Figure 6H).

In contrast, prolonged exposure to PTX significantly decreased hair matrix proliferation, promoted hair matrix keratinocytes apoptosis, and prematurely induced catagen induction (Figure 6, D–F). Long-term ALRN-6924 exposure still abrogated PTX-induced premature catagen (Figure 6F). Furthermore, it protected the HF from PTX-induced cytotoxicity (Figure 6H), DNA damage (increased gH2A.x^+^cells), and pathological EMT (increased vimentin^+^ cells) in the eHFSCs niche (Figure 6, I and J).

**Figure 7:**
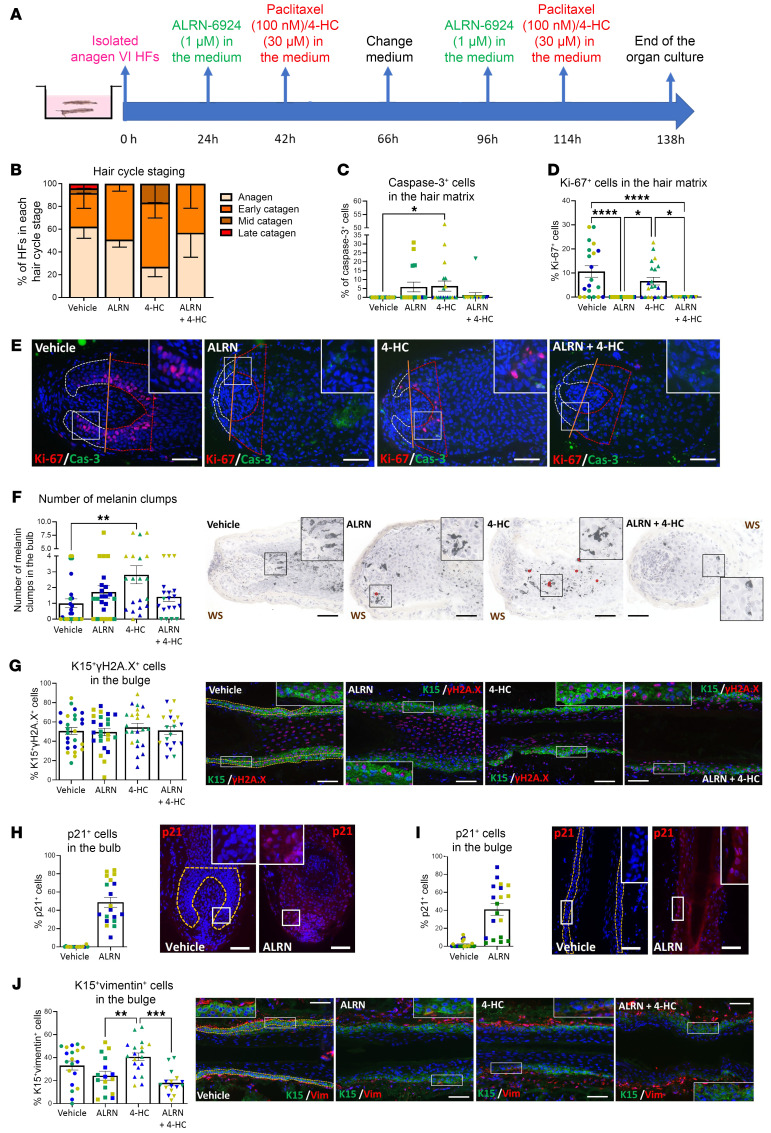
Extended exposure to ALRN-6924 does not promote cytotoxicity and still prevents 4-HC–induced HF damage. (**A**) Experimental design scheme. (**B**–**J**) Quantitative (immuno-)histomorphometry and representative images showing the hair follicle damage induced by 4-HC and the protective role of ALRN-6924 after 6 days of organ culture. (**B**) Hair cycle staging showing the percentage of HFs in each stage. (**C**) Percentage of caspase-3^+^ cells in the hair matrix. (**D**) Percentage of Ki-67^+^ cells in the hair matrix. (**E**) Representative images of Ki-67/caspase-3 double staining. (**F**) Number and representative images of melanin clumps in the defined reference area. (**G**) Percentage and representative image of γH2A.x-positive (DNA damage marker) cells among the total number of K15^+^ cells in the bulge. (**H** and **I**) Percentage of p21^+^ cells in the HF’s bulb (**H**) and bulge (**I**). (**J**) Percentage and representative image of vimentin-positive (marker of pathological EMT) cells among the total number of K15^+^ cells in the bulge. Mean ± SEM; *N* = 16–26 HF/group from 3 donors treated with 4-HC. Student’s *t* test, *P < 0.05, **P < 0.01, ****P < 0.0001. Scale bar 50 μm.

Long-term treatment with 4-HC (Figure 7A) exacerbated catagen induction and apoptosis promotion without altering hair matrix proliferation (Figure 7, B–E), and induced HF dystrophy (melanin clumping) (Figure 7F). It did not further enhance DNA damage in K15^+^ cells in the bulge (Figure 7G), possibly because initially 4-HC–damaged stem cells undergo apoptosis early on, get rapidly eliminated, and are thus no longer detectable. ALRN-6924 again significantly increased the number of p21^+^ cells in the HF bulb and bulge (Figure 7, H and I) and abrogated 4-HC–induced HF dystrophy (melanin clumping) (Figure 7F) and pathological EMT of eHFSCs ex vivo (Figure 7J).

Thus, even prolonged exposure to ALRN-6924 is not HF toxic at the concentration and under the assay conditions tested here and still protects the human HF bulge and matrix from chemotherapy-induced damage ex vivo.

### CIA-protective effects by ALRN-6924 are mediated by p21-driven cell cycle arrest.

**Figure 8:**
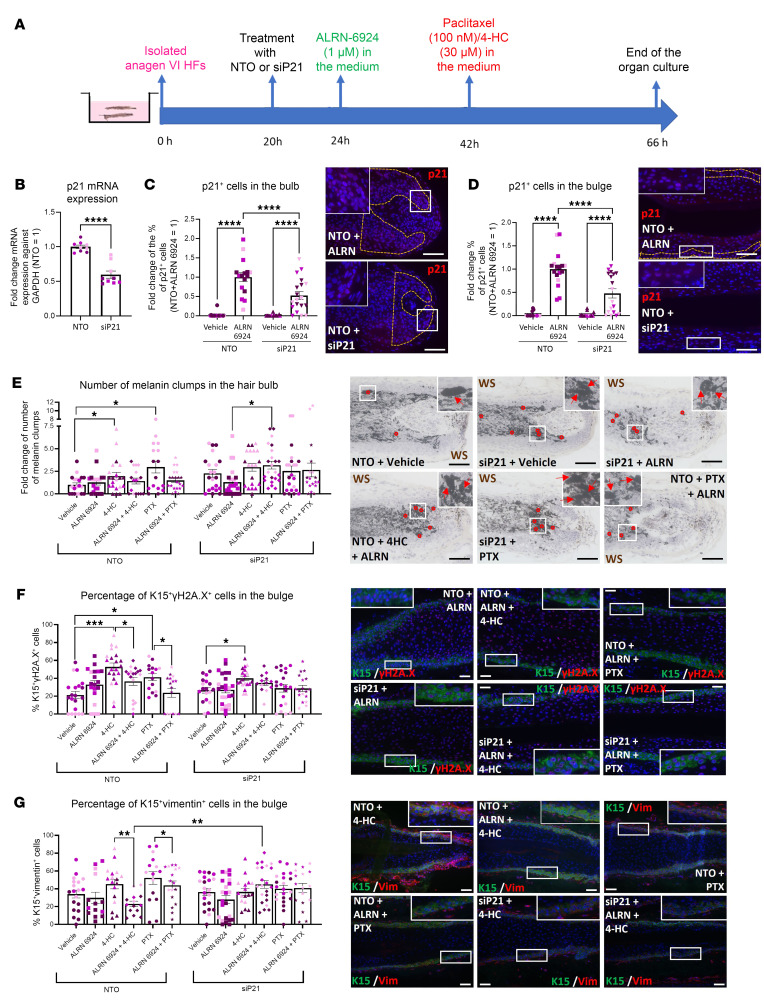
The CIA-protecting effect of ALRN-6924 is promoted via p21-mediated cell cycle arrest. (**A**) Experimental design scheme. (**B**–**G**) Quantitative (immuno-)histomorphometry and representative images showing the hair follicle damage induced by PTX or 4-HC and the effect of ALRN-6924 in the presence or absence of p21 siRNA. (**B**–**D**) p21 transcription levels (**B**) and percentage of p21^+^ cells in the HF’s bulb (**C**) and bulge (**D**). (**E**) Number and representative images of melanin clumps in the defined reference area. (**F** and **G**) Percentage and representative image of γH2A.x-positive (DNA damage marker) (**F**) and vimentin-positive (marker of pathological EMT) (**G**) cells among the total number of K15^+^ cells in the bulge. Mean ± SEM; *N* = 16–25 HF/group from 3 donors treated with ALRN and/or PTX or 4-HC in the presence and absence of p21 siRNA. Student’s *t* test, *P < 0.05, **P < 0.01, ***P < 0.001, ****P < 0.0001. Red arrows: melanin clumps. Scale bar 50 μm.

To probe if the upregulation of p21 is pivotal in the protection mechanism of ALRN-6924, we silenced p21 in HFs treated with either ALRN-6924 alone or in the presence or absence of PTX or 4-HC. Nontargeting oligos (NTO) and p21 siRNA were administered 4 hours before ALRN-6924 treatment, and PTX or 4-HC was added 18 hours later for 24 hours (Figure 8A).

Successful p21 knockdown ex vivo was confirmed by demonstrating a significant decrease in both p21 transcription (qPCR) and protein expression (percentage of p21^+^ cells in the hair bulb and bulge) (Figure 8, B–D) compared with NTO-treated HF. Both PTX and 4-HC induced the expected HF cytotoxicity/melanin clumping, while ALRN-6924 prevented this (Figure 8E). p21 siRNA alone slightly, but not significantly, increased melanin clumping compared with NTO-treated HFs (Figure 8E).

Importantly, p21 silencing abrogated the protective effects of ALRN-6924 against chemotherapy-induced hair matrix dystrophy (melanin clumping) (Figure 8E), eHFSC DNA damage (Figure 8F), and pathological EMT (Figure 8G). This confirms that the chemotherapy-protective effects of ALRN-6924 are p21 dependent.

### Key CIA-protective effects can be reproduced when ALRN-6924 is topically administered to organ-cultured, chemotherapy-treated human scalp skin.

Finally, we asked whether intrafollicular p53/p21 activation and subsequent protection from chemotherapy-induced HF damage can be achieved even when ALRN-6924 is topically administered to healthy, organ-cultured, and chemotherapy-treated human scalp skin, i.e., through an intact epidermal barrier. For this purpose, we used a simple, clinically applicable vehicle containing the well-tolerated ingredients approved for topical minoxidil administration (83, 84). Turbidity studies showed that 10% w/v ALRN-6924 was soluble in a formulation of 30% (v/v) ethanol, 5% (v/v) hydroxypropylcellulose, 50% (v/v) propylene glycol, and 15% deionized water (final ALRN-6924 concentration: 51.8 mM) (Supplemental Figure 4, A and B).

**Figure 9:**
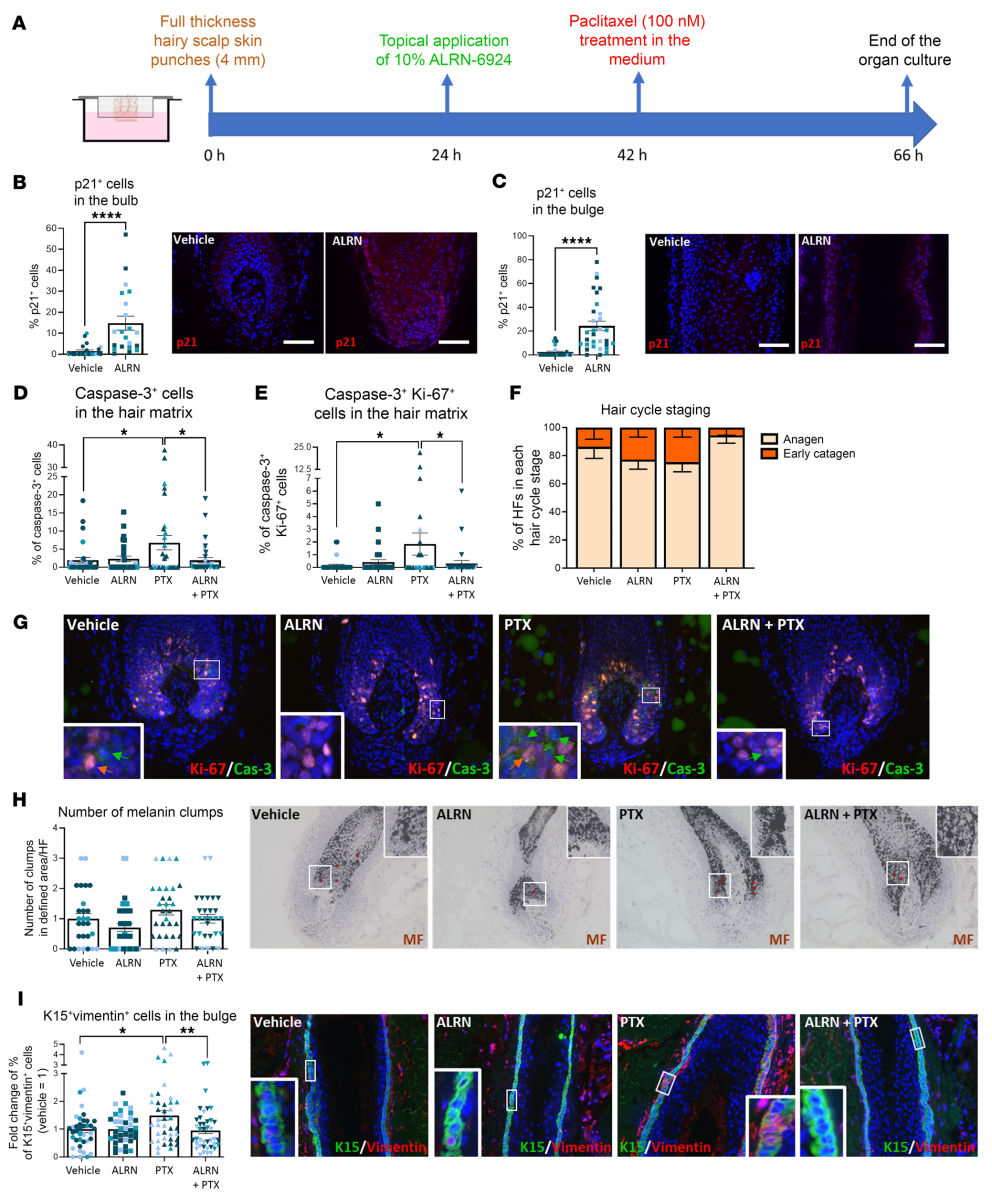
Topically applied ALRN-6924 promotes dystrophic anagen and protects human HF from PTX-induced mitotic catastrophe in scalp skin. (**A**) Experimental design scheme. (**B**–**J**) Quantitative (immuno-)histomorphometry and representative images illustrating the HF damage induced by PTX as well as the effect of topically applied ALRN-6924 on hairy scalp skin. (**B** and **C**) Percentage of p21^+^ cells in the HF’s bulb (**B**) and bulge (**C**). (**D** and **E**) Percentage of apoptotic (caspase-3^+^) cells in the hair matrix (**D**) and the number of caspase-3^+^Ki-67^+^ cells showing the “mitotic catastrophe” in the hair matrix (**E**). (**F**) Hair cycle staging, showing the percentage of HFs in each stage. (**G**) Representative image of Ki-67/caspase-3 double staining. (**H**) Number and representative images of melanin clumps in the defined reference area. (**I**) Percentage and representative image of vimentin-positive (marker of pathological EMT) cells among the total number of K15^+^ cells in the bulge. Mean ± SEM; *N* = 33–36 HF/group from 3 donors treated with ALRN-6924 and/or PTX. Student’s *t* test, *P < 0.05, **P < 0.01, ****P < 0.0001. Green arrows: caspase-3^+^ cells. Orange arrows: Ki-67^+^caspase-3^+^ cells.

When only 2 μL of the topical ALRN-6924 formulation was applied to 4 mm skin fragments that were organ-cultured at the air-liquid interface on a cell strainer (Figure 9A) (57), 18 hours before adding PTX to the culture medium, upregulated p21 expression was detected in the HF bulb and bulge (Figure 9, B and C). This demonstrates that topically applied ALRN-6924 reaches the anagen hair bulb in the chosen HF-targeting vehicle, despite the very low applied volume.

In this assay, PTX induced measurable hair bulb damage (apoptosis, “mitotic catastrophe,” melanin clumping) but did not alter HF cycling (Figure 9, D–H) and the number of apoptotic CD31^+^ cells (Supplemental Figure 4C). Topically applied ALRN-6924 protected K15^+^ eHFSCs from PTX-induced EMT (Figure 9I) and slightly, but not significantly, also from apoptosis (Supplemental Figure 4D). Consistent with the results from the “systemic” ALRN-6924 treatment experiments reported above, significant protection from PTX-induced apoptosis, mitotic catastrophe, and bulb cytotoxicity was also observed when only a very small volume of ALRN-6924 was topically applied to human scalp skin ex vivo prior to PTX treatment (Figure 9, D–I).

## Discussion

The current study shows that both “systemic” and topical treatment of HFs and scalp skin with a clinical-stage p53-activating peptide (ALRN-6924) induces p21-mediated cell cycle arrest, as confirmed by increased p21 transcription and protein expression and decreased transcription of Ki-67 and CDK1. Treatment with ALRN-6924 prior to chemotherapy protects healthy human HFs and their eHFSCs against multilevel toxicity primarily mediated at the level of cell cycle arrest. Given the documented selectivity of ALRN-6924 for TP53-WT (40, 50), this “stapled peptide” promises to selectively protect healthy human cell populations against adverse effects of chemotherapy in the ~50% of cancer patients with p53-mutant malignant tumors (52–54). This protection might also extend to chemotherapy-sensitive extracutaneous stem cells and other rapidly proliferating cell populations, such as in mucosal epithelia, bone marrow, and testis, i.e., other primary target tissues of chemotherapy-induced toxicity (50, 85–87).

Even topical ALRN-6924 has chemotherapy-protective effects for healthy eHFSCs (Figure 9). Under clinical conditions, topical application of ALRN-6924 further limits the risk of conferring survival benefits to extracutaneous cancer cells and of potential systemic adverse effects, and could be combined with CIA-protective cooling caps (18–20), whose efficacy it may enhance. Yet, while these caps can only protect the scalp, topical ALRN-6924 can be applied to all CIA-affected facial and other cosmetically important skin regions. Extensive safety data on systemically administered ALRN-6924 (i.v.) are available from clinical trials, with mild nausea and fatigue recognized as primary systemic side effects (41). Yet residual systemic levels after short-term therapy with topical ALRN-6924 would be expected to be too low to elicit these adverse effects.

Our ex vivo results (Figure 6E and Figure 7C) suggest that ALRN-6924 may be administered for an extended period if clinically indicated. However, in routine oncological practice, chemoprotective ALRN-6924 would only be administered shortly before, during, and immediately after chemotherapy, i.e., for less than 24 hours, which further reduces the risk of adverse effects, both in the HF and extracutaneous tissues.

Since ALRN-6924 targets primarily highly proliferative cells and promotes cell cycle arrest via WT p53–mediated transient upregulation of p21 (40, 47–49), its chemoprotective effects are most likely p53 mediated. In fact, only cycling (nonquiescent) cells are susceptible to pharmacological activation of p53 and upregulation of p21. For example, in bone marrow, only 5%–20% of cells are nonquiescent (88, 89), and systemic ALRN-6924 upregulates p21 in only 3%–7% of bone marrow cells in healthy human volunteers (90). That p21 is activated in all HF cells is not an expected result; instead, the desired outcome is to arrest only the subpopulations of proliferating stem and hair matrix cells to protect them from chemotherapy-induced toxicity. Our data show that the main protection mechanism of ALRN-6924 is p21 dependent, since p21 silencing ex vivo abrogates the chemoprotective effects of ALRN-6924 (Figure 8).

Besides confirming that PTX and 4-HC damage both massively proliferating keratinocytes in the anagen hair matrix and relatively quiescent eHFSCs (9, 15, 30), we show here that PTX causes not only apoptosis, “mitotic catastrophe,” and DNA damage but also pathological EMT in the bulge of human scalp HFs, thus further depleting their crucial epithelial stem cell niche and compromising their regenerative capacity (33) — a persuasive explanation for the alarmingly high incidence of permanent hair loss under taxane therapy (26, 29, 91, 92).

That a cell cycle–arresting peptide protects eHFSCs from pathological EMT (Figure 5, F and G; Figure 6J; Figure 7J; Figure 8G; and Figure 9I) was unexpected. TGF-β2, a well-known EMT promoter (93, 94), including in the human bulge (80), was upregulated by PTX, but ALRN-6924 did not prevent this increase (also, ALRN-6924 alone did not affect TGF-β2 gene expression) (Figure 3). This suggests a TGF-β2–independent mechanism of EMT inhibition. Interestingly, in epithelial cancer cells, cell cycle arrest can promote EMT (95–98), whereas p53 inhibits vimentin expression and EMT by transcriptionally activating the miR200 family, which suppresses ZEB-1/-2 expression (99, 100). Therefore, ALRN-6924 may render eHFSCs less sensitive to chemotherapy-induced EMT by cell cycle arrest. If confirmed, this peptide may also be beneficial in scarring alopecias like frontal fibrosing alopecia and lichen planopilaris, characterized by pathological EMT of eHFSCs (28, 32, 80).

We chose a strictly human HF and scalp skin organ culture design for this study, since mouse CIA models could be misleading, e.g., due to major differences in hair cycle dynamics between murine and human HFs (101, 102). Moreover, the bulge epithelium of murine HFs reportedly harbors nestin^+^ cells that can stimulate hair growth (103) and may provide some protection against CIA, presumably via the HF vasculature (77). In contrast, the main targets of chemotherapy-induced HF toxicity in humans are hair matrix keratinocytes and eHFSCs (11, 12, 15, 16, 30, 76). Yet in human HFs, nestin transcription is restricted to the HF mesenchyme (103, 104). “nestin”-like immunoreactivity can be observed in the bulge outer root sheath, where it colocalized with K15 *in cryosections* (Supplemental Figure 5), and in the perifollicular dermis, which is not relevant to the current context. However, this “nestin”-like immunoreactivity is an artifact, since it does not correspond to nestin mRNA expression within the HF epithelium (112, 113). Indeed, in HOPE-fixed sections, no nestin immunoreactivity is detected in human bulge epithelium (103). Furthermore, contrary to a previous report (105) “nestin”-pseudopositive cells colocalized perfectly with K15^+^ cells. Yet, given the association of nestin^+^ cells with human skin appendages, namely eccrine glands (106–108) and their astounding regenerative properties, including in experimentally wounded human skin (109), it is worth investigating whether cell therapy with autologous nestin^+^ cells can promote HF repair or regeneration after chemotherapy-induced damage, in addition to chemoprevention by topical ALRN-6924.

Finally, hair regrowth following CIA critically depends on the specific HF cycling response to chemotherapy-induced damage: the reparative “dystrophic anagen” response pathway, which is clinically associated with a reduced degree of acute hair loss, versus the “dystrophic catagen” pathway, which is associated with the most severe acute CIA but also with the fastest hair regrowth (4, 8, 11, 33). Our finding that ALRN-6924 promotes a mild version of “dystrophic anagen” and inhibits “dystrophic catagen” ex vivo predicts that, in an in vivo setting, pretreatment with ALRN-6924 will reduce chemotherapy-induced anagen effluvium and thus acute CIA (4, 25, 33). Instead, its profound eHFSC-chemoprotective effects suggest that ALRN-6924 may also reduce the development of permanent CIA. Taken together with its unique selectivity for healthy cells expressing WT p53 and its topical efficacy, this renders ALRN-6924 a promising candidate CIA protectant in patients with p53-mutant tumors and warrants systematic study in clinical trials.

## Methods

### Sex as a biological variable.

Since CIA affects both sexes equally (110), temporal and occipital human scalp skin was obtained from either male or female healthy donors undergoing routine facelift surgery or hair transplant in the United States or Germany. In this study, samples from both sexes were used. Findings are expected to apply to both sexes.

### Human samples.

Terminal anagen VI scalp HFs were microdissected from scalp skin samples as described (111, 112). For full-thickness scalp skin studies, 4 mm skin punches were sampled for systemic or topical application of ALRN-6924. No sample size calculation was performed due to the limited availability of human tissue samples. A total of 23 donors were enrolled in the different experiments reported here (13 male and 10 female; age range, 25–60 years; mean age, 47 years). Human scalp samples were obtained 1 day after facelift or hair transplantation surgery.

### Human skin and HF organ culture.

Full-length terminal scalp HFs in anagen VI (63) were microdissected to remove all perifollicular tissue with the sole exception of the HF’s dermal sheath (15). HFs or 4 mm full-thickness scalp skin punches were cultured at 37°C with 5% CO_2_ in serum-free William’s E minimal media (Gibco, Thermo Fisher Scientific) supplemented with 2 mM of l-glutamine (Gibco, Thermo Fisher Scientific), 10 ng/mL hydrocortisone (Sigma-Aldrich), 10 μg/mL insulin (Sigma-Aldrich), 1% amphotericin B, and 1% penicillin/streptomycin mix (Gibco, Thermo Fisher Scientific) as previously described (57, 113–118). Investigators who wish to explore the HF-protective effects of candidate CIA protectants in the presence of serum can employ an instructive alternative scalp skin organ culture model (119).

After 24 hours, HFs or 4 mm full-thickness scalp skin punches were randomly assigned to experimental groups, the medium was replaced, and samples were treated with vehicle (0.1% DMSO) or “systemic” ALRN-6924 (1 μM) (42). At 18 hours after the medium was changed, either 100 nM PTX or 30 μM 4-HC was added alone or in combination with ALRN-6924 (11, 15, 30). HFs were embedded after 24 hours of treatment into OCT compound (Thermo Fisher Scientific) and snap-frozen in liquid nitrogen for qIHM (see below). This study design mimicked the clinical HF exposure time in breast cancer patients to PTX (mean residence time: 4–6 hours) or cyclophosphamide/4-HC treatment (10–13 hours) (120) and the half-life of ALRN-6924 (p21^+^ cells are detected up to 20 hours after compound administration) (41, 43). Moreover, the CDK4/6 inhibitor palbociclib protects transit-amplifying and stem/progenitor cells’ PTX cytotoxicity through G1 arrest within this window (15). For the 6-day assay, ALRN-6924 and the chemotherapy agents were applied twice, as described above, on days 2 and 4 and days 3 and 5, respectively.

p21 silencing was performed by transfecting anagen HFs ex vivo with 1 μM of human p21-specific siRNA probe (ON-TARGETplus Human CDKN1A [Entrez gene number 1026] siRNA - SMARTpool, 5 nmol [siP21; Horizon Discovery Biosciences Limited, catalog L-003471-00-0005]) or NTO (ON-TARGET plus Non-targeting Pool, 20 nmol Horizon Discovery Biosciences Limited, catalog D-001810-10-20) using Lipofectamine RNAiMAX (Horizon Discovery Biosciences Limited) following the manufacturer’s instructions (121–123). NTO or p21 siRNA was applied 4 hours prior to ALRN-6924 treatment, followed by PTX or 4-HC administration (Figure 8A).

For the topical application of ALRN-6924, 4 mm skin punches were cultured at the air-liquid interface on a gelatin sponge, and after 24 hours of preincubation, 2 μL of 10% ALRN-6424 was topically applied (just 2 μL suffices as volume for topical application in human skin organ culture) (57, 126), thus avoiding leakage into the medium. After 18 hours, the medium was changed and PTX was added.

### qRT-PCR.

Total RNA was isolated from whole microdissected HFs using PicoPure RNA Isolation Kit (Applied Biosystems, Thermo Fisher Scientific) (123, 124), and genomic DNA was eliminated by DNase I treatment, RNase-free (QIAGEN). RNA purity and concentrations were determined using the NanoDrop ND-1000 assay (Thermo Fisher Scientific). Reverse transcription of the RNA into cDNA was performed using the TetrocDNA Synthesis Kit (Bioline-Meridian Bioscience), according to the manufacturer’s instructions. The RNA concentrations were adjusted (between 50 and 500 nM) to have the same amount of RNA among the different groups to allow further quantification and comparison. qRT-PCR was run in triplicate on the CFX Connect thermal cycler and detection system (Bio-Rad) using TaqMan Fast Advanced Master Mix and Gene Expression Assay probes (Id: Hs02758991_g1 for GAPDH, Hs00355782_m1 for Cdkn1a/p21, Hs00180269_m1 for BAX, Hs04986394_s1 for Bcl-2, Hs00938777_m1 for CDK1, Hs04260396_g1 for MKI67, Hs01547656_m1 for IGF-1, and Hs00234244_m1 for TGF-β2, Applied Biosystems, Thermo Fisher Scientific). The transcript levels were normalized to GAPDH using the ΔΔCT method (71, 123, 124).

### Immunofluorescence microscopy and histochemistry.

OCT-embedded HFs and skin punches were cryosectioned (6 or 7 μm, respectively) and processed for immunofluorescence microscopy following our established protocols (71, 125). K15^+^ epithelial stem cells in the basal layer of the bulge (34, 59, 126, 127) were examined, since these are primarily affected by PTX (12, 15, 30). In human scalp HFs, cells that both transcribe and translate Nestin are restricted to the bulge mesenchyme and are absent from bulge epithelium (103). There is no evidence that the bulge mesenchyme or perifollicular vasculature play a critical role in human CIA, whereas K15^+^ stem cells in the bulge epithelium are highly susceptible to chemotherapy-induced damage (12, 15, 30). Therefore, K15^+^ eHFSCs are the key stem cell population for investigation in this context.

We also performed double-immunostaining of untreated day 0 skin for nestin and K15. In summary, cryosections were fixed in 4% paraformaldehyde (PFA) in PBS, then blocked with 5% BSA and 0.3% Triton X-100 in PBS. Primary antibodies for nestin (mouse, clone 10C2, Merck, 1:250) and K15 (guinea pig polyclonal, Progen, 1:500) were added to sections and incubated overnight. The following day, goat anti-mouse Alexa Fluor 555, A-21422, Invitrogen, Thermo Fisher Scientific, 1:400, and goat anti–guinea pig Alexa Fluor 488, A-11073, Invitrogen, Thermo Fisher Scientific, were added for 45 minutes at room temperature (RT).

The percentage of apoptotic/proliferating cells in the hair matrix or among K15^+^ eHFSCs was assessed by triple immunostaining for Ki-67, cleaved caspase-3, and K15. Briefly, primary antibodies for cleaved caspase-3 (rabbit polyclonal, 9661S, Cell Signaling Technology, 1:400) and Ki-67 (mouse, clone 8D5, Cell Signaling Technology, 1:800) were incubated overnight at 4°C, followed by secondary antibodies (goat anti-rabbit Alexa Fluor 647, A-21245, Invitrogen, Thermo Fisher Scientific, 1:400, and goat anti-mouse Alexa Fluor 555, A-21422, Invitrogen, Thermo Fisher Scientific, 1:400, correspondingly) for 45 minutes at RT. After a blocking step using 10% BSA in PBS, the K15 antibody (mouse anti-CK15 FITC-conjugated; NBP2-54462F, Novus Biologicals, Bio-Techne; 1:200) was incubated for 1 hour at 37°C.

For evaluating p21, γH2A.x (15), vimentin (30, 80), or K85 (70) expression, and to detect apoptotic endothelial cells (caspase-3/CD31) (109), HF or scalp skin cryosections were incubated with the corresponding primary antibody (mouse anti-p21, 556431, BD Pharmingen, 1:200; or rabbit anti-γH2A.x, 2577S, Cell Signaling Technology, 1:500; or mouse anti-vimentin, MAB3400, Millipore, 1:200; or guinea pig anti-K85, GP-HHB5, Progen, 1:1,000; or mouse anti-CD31, M082329-2, Agilent, 1:100 and rabbit anti–caspase-3, 9661S, Cell Signaling Technology, 1:400, respectively) at 4°C overnight. Secondary antibody (goat anti-mouse Alexa Fluor 555, A-21422, Invitrogen, Thermo Fisher Scientific; 1:400 or goat anti-rabbit Alexa Fluor 555, A-21429, Invitrogen; Thermo Fisher Scientific, 1:400; goat anti–guinea pig Alexa Fluor 594, A-11076, Invitrogen, Thermo Fisher Scientific, 1:400; goat anti-rabbit Alexa Fluor 488, A-11073, Thermo Fisher Scientific) was incubated at RT for 45 minutes. γH2A.x and vimentin immunostaining was followed by incubation with K15 antibody (mouse anti-CK15 FITC-conjugated; NBP2-54462F, Novus Biologicals, Bio-Techne; 1:200) for 1 hour at 37°C. Finally, slides were embedded with DAPI/Fluoromount-G (Electron Microscopy Sciences) to visualize nuclei.

To investigate HF pigmentary abnormalities, cryosections were fixed in 4% PFA for 10 minutes at RT and processed following the manufacturer’s procedure (Warthin-Starry Stain Kit, Abcam) (128). Glycogen content and basal membrane integrity were assessed using PAS staining as described (67).

### qIHM.

Images were captured using a Keyence fluorescence microscope (BZX810, Keyence Corporation) with a constant exposure time. Analysis was performed using NIH ImageJ software (9, 12, 68). The number of cells undergoing apoptosis (cleaved caspase-3^+^), proliferation (Ki-67^+^), DNA damage (γH2AX^+^), or pathological EMT (vimentin^+^) was counted in the bulge area among the total number of K15^+^ cells. Immunoreactivity for K15 was evaluated in the bulge. The number of proliferative (Ki-67^+^) or apoptotic (cleaved caspase-3^+^) cells in the hair matrix was counted among the total number of cells (DAPI-positive nuclei) in defined reference areas and expressed as a percentage (63, 71). The percentage of apoptotic endothelial cells (caspase-3^+^CD31^+^ cells) was counted around the HFs within a 100 μm wide area from the HFs’ basal membrane. Hair cycle staging and scoring were performed as described (63). Melanin clumping, glycogen, and basal membrane integrity were evaluated as described (67, 68).

### Statistics.

All data are expressed as fold-change of mean or mean ± SEM and were analyzed either by 1-way ANOVA or Kruskal-Wallis test and Dunn’s multiple comparisons test as post hoc test when more than 2 groups were compared, or Student’s 2-tailed *t* test or Mann-Whitney *U* test when 2 groups were compared (GraphPad Prism 6 and 9), after performing d’Agostino and Pearson omnibus normality tests. *P* < 0.05 was regarded as significant. The number of HFs and donors analyzed for each dataset is stated in the corresponding figure legend. Given the biological diversity among individual HFs, which represent distinct mini-organs with significant interfollicular differences in almost every hair biology readout parameter (129), the number of HFs was taken as *N*, rather than the number of donors. Moreover, human scalp HFs show mosaic, nonsynchronized HF cycling (102). Therefore, it is biologically appropriate to consider each HF in the ex vivo model used here as a biological replicate (71, 121, 123, 130–132). To probe interindividual data reproducibility between donors, a minimum of 3 donors was examined, as is standard practice in the field.

### Study approval.

The use of anonymized, discarded human tissues was considered nonhuman research and exempted under the Section 15 of the Professional Code of Conduct of the Berlin Medical Association (Eth-MZ-24-020) and Section 15 of the Bavarian Professional Code of Conduct for Doctors (2024-1066) and under 45 CFR46.101.2 by the Institutional Review Board of the University of Miami Miller School of Medicine.

### Data availability.

All data necessary to evaluate the conclusions in the paper are presented in the article, in the supplement, or in the Supporting Data Values, and the original data sets and related data generated during the study are available from the corresponding author upon reasonable request.

## Author contributions

RP conceived and supervised, and JC, JG, and RP designed the study. JG, JC, KL, TS, TGG, DAA, and SDV performed experiments and/or analyzed data, which JG, TS, TGG, AA, SDV, KL, TCW, UK, JRF, RK, WF, RPA, DAA, MA, JC, and RP interpreted. UK designed the topical vehicle. RP and JC wrote the manuscript, with significant editorial input from JG, DAA, MA, and TCW. JRF, RK, WF, and RPA provided all human tissue samples. All authors read, edited, and approved the final manuscript.

## Conflict of interest

AA and MA were employees and shareholders of Aileron Therapeutics, Inc. RP, JC, KL, and JG are employees of and UK is a consultant of CUTANEON - Skin & Hair Innovations GmbH.

## Funding support

This work is the result of NIH funding, in whole or in part, and is subject to the NIH Public Access Policy. Through acceptance of this federal funding, the NIH has been given a right to make the work publicly available in PubMed Central.

- Aileron Therapeutics.
- CUTANEON - Skin & Hair Innovations.
- R21CA277418 (NIH/NCI) to RP and TCW.
- Frost Endowed Scholarship/Investigatorships from the Department of Dermatology, University of Miami, to RP and TCW.

## Supplementary Material

Supplemental data

Supporting data values

## Version 1 05/01/2026

Electronic publication
