# Supplementary material for: Transient p53/p21 activation selectively protects healthy human hair follicles and their stem cells from chemotherapy
Source: J Clin Invest. 2026 May 1;136(9):e174447. doi: 10.1172/JCI174447 (PMC13132392; doi:10.1172/JCI174447)
Supplement: Supplemental data [file jci-136-174447-s349.pdf]

Supplemental figures

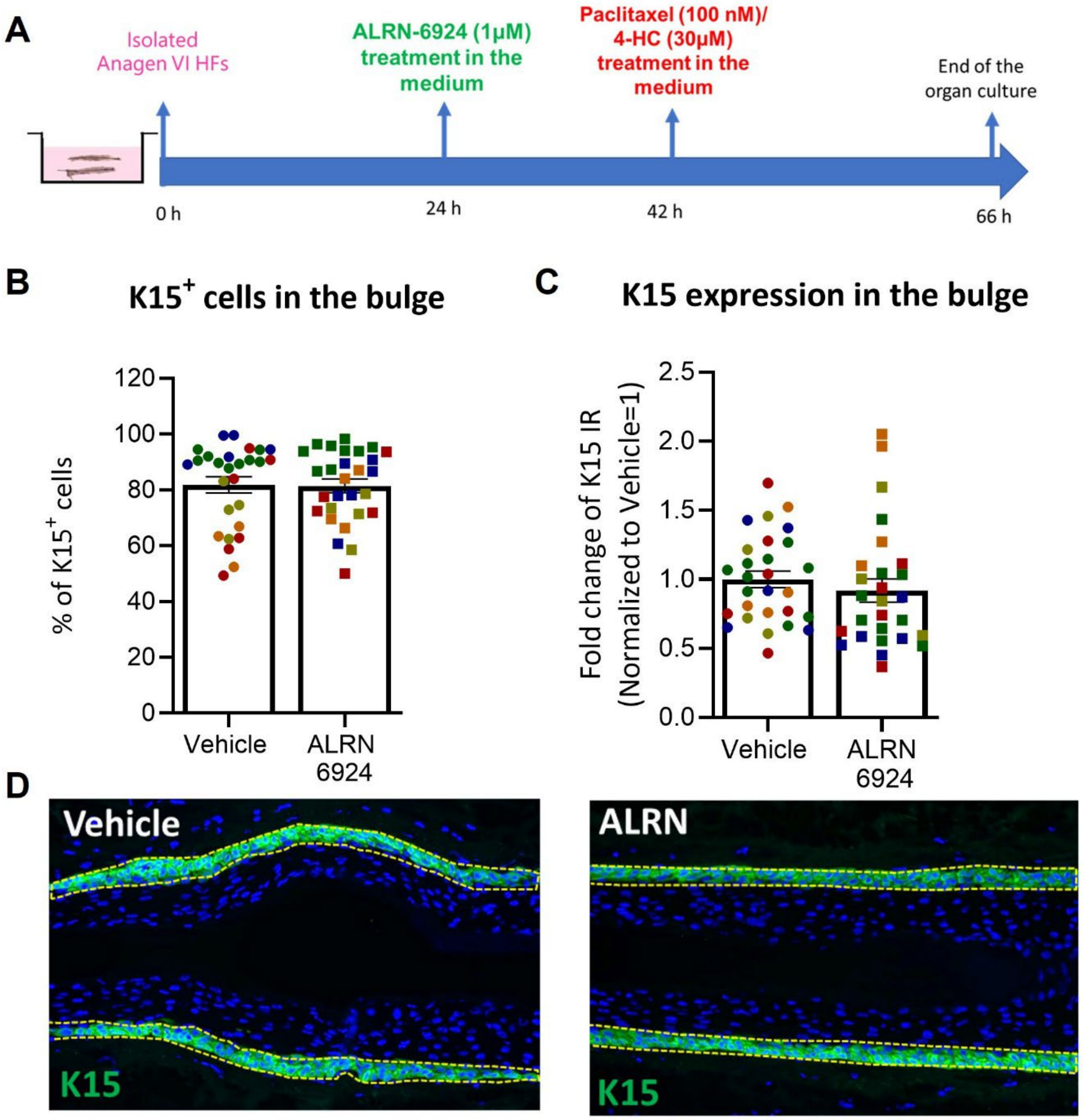

Supplemental Figure 1: Effect of ALRN-6924 on K15^{+} eHFSC in the microdissected hair follicle.

(A) Experimental design scheme. (**B-C**) Percentage (**B**) and immunoreactivity (**C**) of K15^{+} cells in the bulge. (**D**) Representative images of K15 staining. Mean (±) SEM; N= 20-29 HF/group from 5 donors. Student’s t-test.

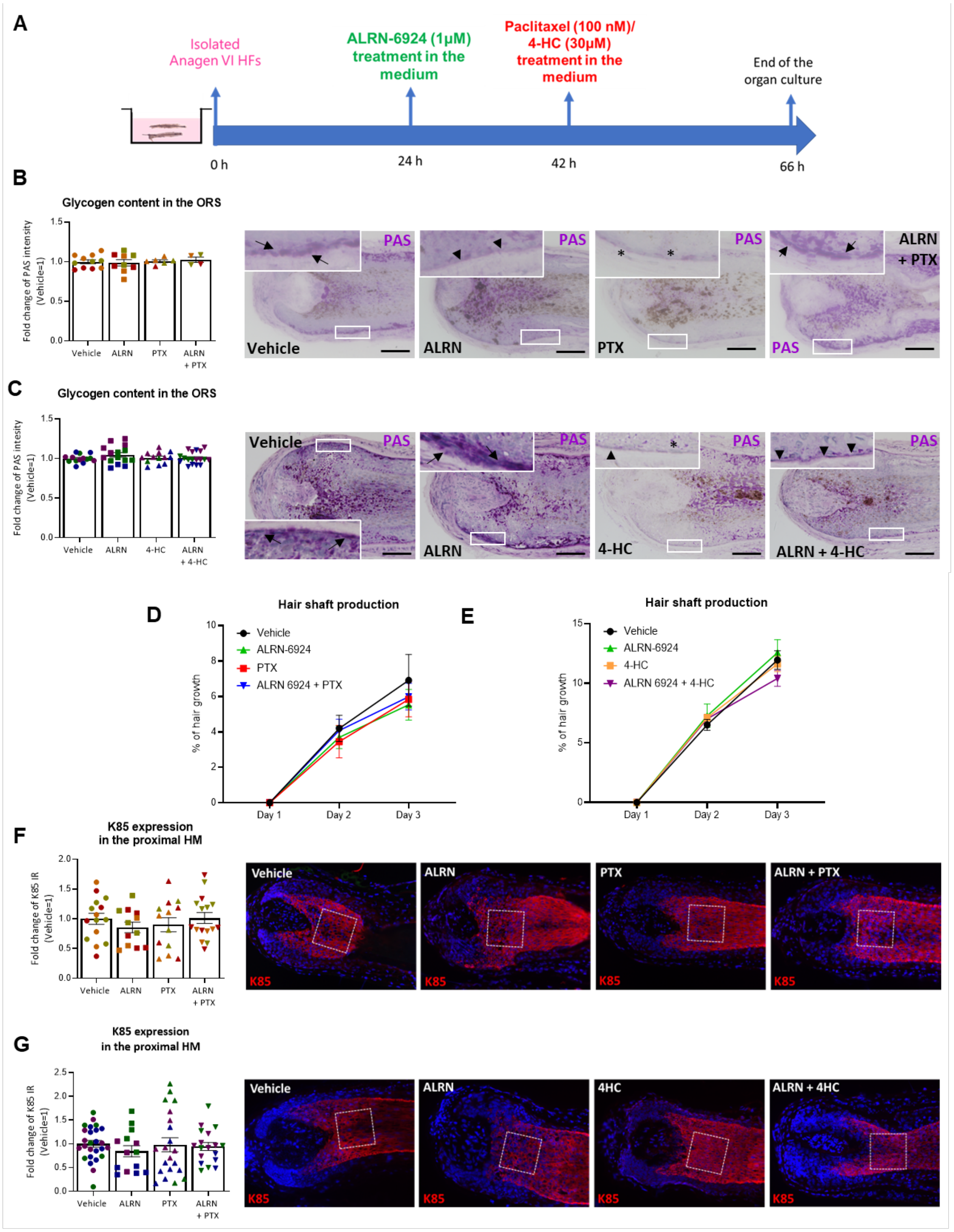

Supplemental Figure 2: Effect of chemotherapy and ALRN-6924 on HFs integrity and growth.

(A) Experimental design scheme. (**B-C**) Quantitative (immuno-)histomorphometry and representative images showing PAS staining for detecting glycogen content and basement

membrane integrity in HFs treated with ALRN-6924 alone or in combination with PTX (**B**) or 4-HC (**C**). Mean (±) SEM; N= 4-14 HF/group from 3 donors. Student's t-test, n.s. (**D-E**) Hair shaft production during the 3 days of organ culture of HFs treated with ALRN-6924 alone or in combination with PTX (**D**) or 4-HC (**E**). Mean (±) SEM; N= 14-15 HF/group from 3 donors. Student's t-test, n.s. (**F-G**) Immunoreactivity and representative images of K85 expression in HFs treated with ALRN-6924 alone or combined with PTX (**B**) or 4-HC (**C**). Mean (±) SEM; N= 12-25 HF/group from 3 donors. Student's t-test, n.s.

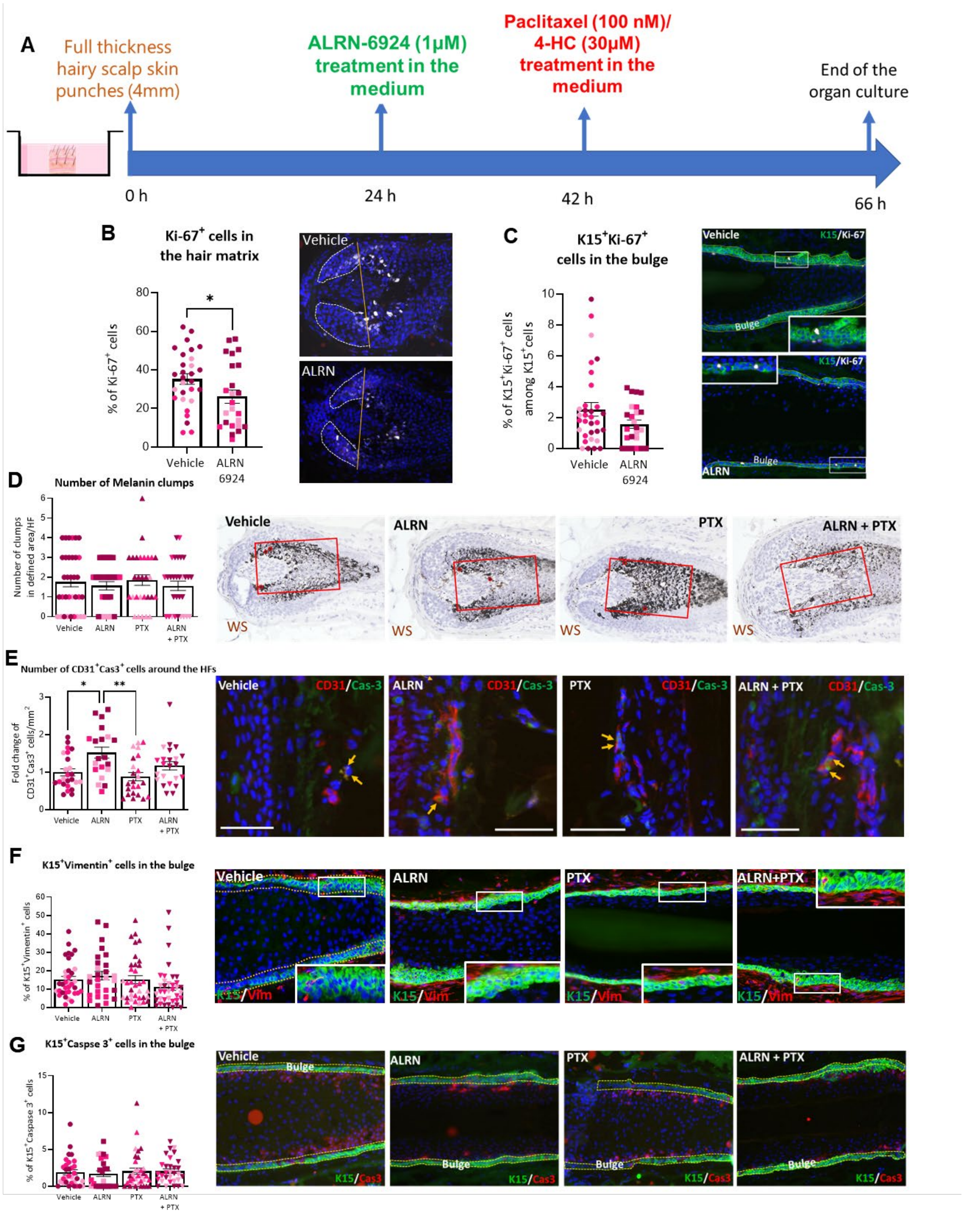

Supplemental Figure 3: Efficacy of ALRN-6924 and absence of PTX-induced hair follicle damage in human scalp skin.

(A) Experimental design scheme. (**B-C**) Quantitative (immuno-)histomorphometry and representative images showing cell cycle arrest mediated by ALRN-6924. Percentage of Ki-67$^+$ cells in the hair matrix (**C**) and K15$^+$/Ki-67$^+$ cells in the bulge. Mean (±) SEM; N= 24-32 HF/group from 3 donors treated with PTX. Student's t-test. (**D**) Number and representative images of melanin clumps in the defined reference area. Mean (±) SEM; N= 28-35 HF/group from 3 donors treated with PTX. Student's t-test. (**E**) Percentage and representative picture of apoptotic endothelial cells (CD31$^+$Caspase 3$^+$) around the HFs. Mean (±) SEM; N= 20-24 HF/group from 3 donors treated with PTX. Student's t-test, *$p<0.05$, **$p<0.001$. (**F-G**). Percentage and representative images of K15$^+$Vimentin$^+$ (**F**) or K15$^+$Caspase 3$^+$ (**G**) cells among the total number of K15$^+$ cells in the bulge. Mean (±) SEM; N= 28-35 HF/group from 3 donors treated with PTX. Student's t-test.

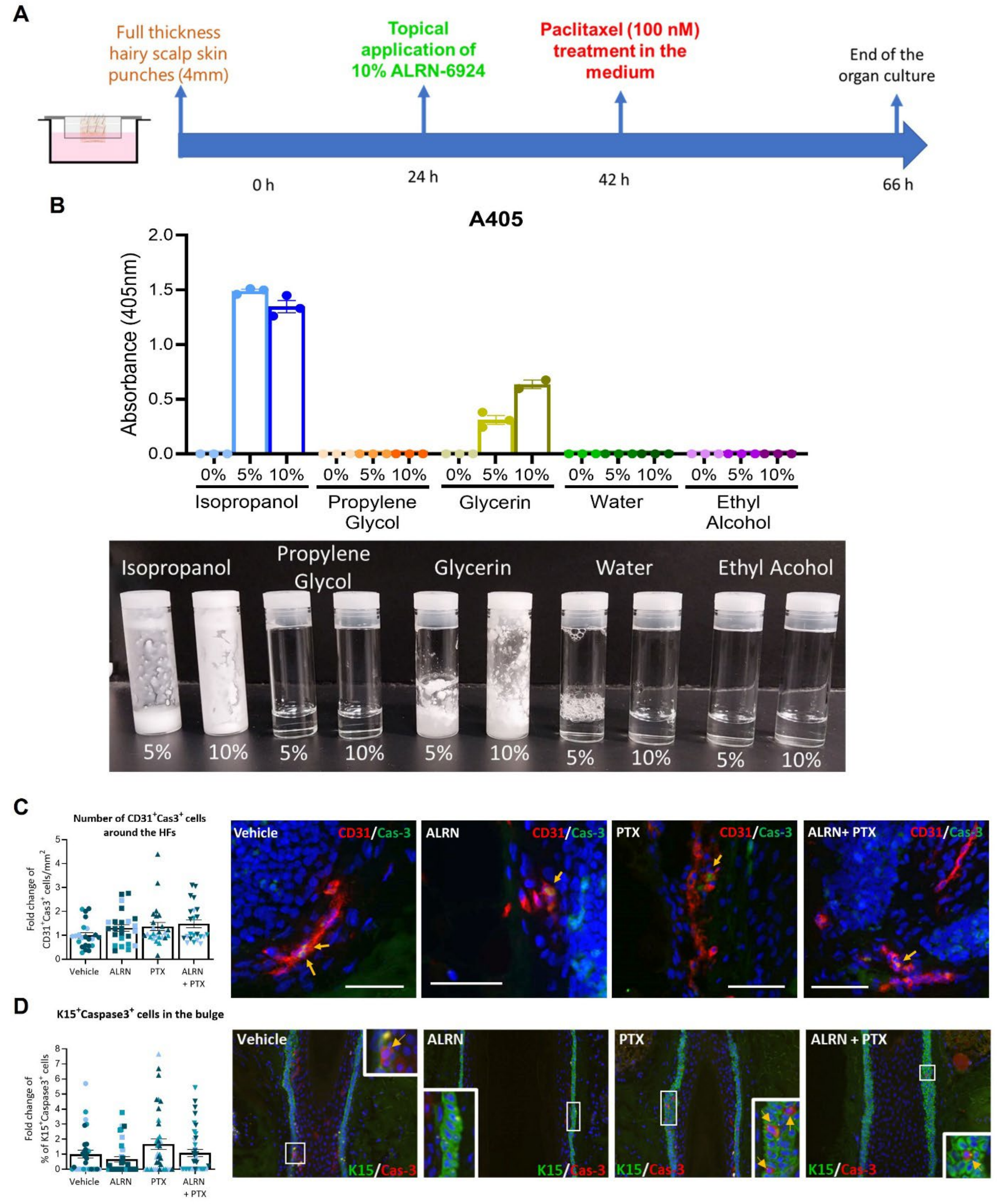

Supplemental figure 4: Turbidity assay results for ALRN-6924 in media for topical application.

(A) Experimental design scheme. (**B**) Turbidity test showing the solubility of 5% and 10% w/v concentrations (25.9 and 51.8 mM) of ALRN-6924 in different topical application media. UV

Absorbance as a proxy for turbidity read at 405 mm. (**C**) Percentage and representative picture of apoptotic endothelial cells ($CD31^{+}Caspase\ 3^{+}$) around the HFs. Mean (±) SEM; N= 23-25 HF/group from 3 donors treated with PTX. Student's t-test. (**D**) Percentage and representative image of apoptotic (Caspase 3^{+}) $K15^{+}$ cells among the total number of $K15^{+}$ cells in the bulge. Mean (±) SEM; N= 30-39 HF/group from 3 donors treated with PTX. Student's t-test

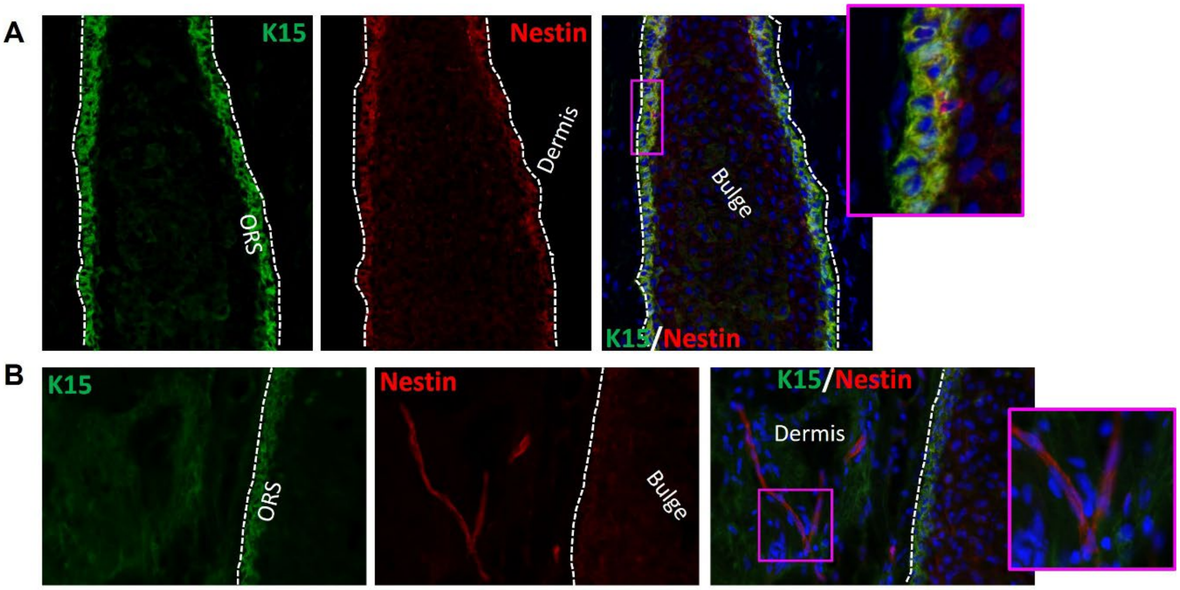

Supplemental Figure 5: (A-E) Nestin and K15 expression in human HFs. (**A**) Positive staining for K15 and nestin in the bulge epithelium of uncultured human hairy scalp skin. (**B**) Positive staining for K15 in the bulge epithelium of uncultured human hairy scalp skin, while nestin expression is restricted to perifollicular dermis, which seems to correspond to endothelial cells**.**
